# Research on the Laser Scattering Characteristics of Three-Dimensional Imaging Based on Electro–Optical Crystal Modulation

**DOI:** 10.3390/mi15111327

**Published:** 2024-10-30

**Authors:** Houpeng Sun, Yingchun Li, Huichao Guo, Chenglong Luan, Laixian Zhang, Haijing Zheng, Youchen Fan

**Affiliations:** 1 Graduate School, Space Engineering University, Beijing 101416, China; sunhoupeng@hgd.edu.cn (H.S.); lclzdfcs@hgd.edu.cn (C.L.); 2 Department of Electronic and Optical Engineering, Space Engineering University, Beijing 101416, China; zhanglaixian@126.com (L.Z.); 3120120251@bit.edu.cn (H.Z.); 3 School of Space Information, Space Engineering University, Beijing 101416, China; love193777@sina.com

**Keywords:** laser scattering characteristics, electro–optical crystal, laser 3D imaging simulation model, hybrid BBO–Firefly algorithm, BRDF model

## Abstract

In this paper, we construct a laser 3D imaging simulation model based on the 3D imaging principle of electro–optical crystal modulation. Unlike the traditional 3D imaging simulation method, this paper focuses on the laser scattering characteristics of the target scene. To accurately analyze and simulate the scattering characteristic model of the target under laser irradiation, we propose a BRDF (Bidirectional Reflectance Distribution Function) model fitting algorithm based on the hybrid BBO–Firefly model, which can accurately simulate the laser scattering distribution of the target at different angles. Finally, according to the fitted scattering characteristic model, we inverted the target imaging gray map. We used the laser 3D imaging restoration principle to reconstruct the 3D point cloud of the target to realize the laser 3D imaging of the target.

## 1. Introduction

Laser 3D imaging technology has many applications in target observation and recognition [1,2,3,4,5,6]. Three-dimensional imaging based on electro–optical (EO) crystal modulation is a new imaging technology that can quickly obtain the size and structure information of the target and has the advantage of not being affected by light and atmosphere [7,8]. Compared with the traditional laser 3D imaging technology, the 3D imaging method based on EO crystal modulation uses an industrial camera with high quantum efficiency, large area array, and high sensitivity to replace ICCD (Intensified Charge-Coupled Device) as the imaging detector [9,10,11,12,13,14]. To achieve target imaging at a specific distance, the EO effect of the crystal is used to achieve a gating function with a narrow pulse width with a high repetition rate. In 2016, Professor Jo designed a three-dimensional imaging system based on EO crystal modulation based on the sub-focal plane [15]. The system uses the modulation phase information of the crystal to the flight time of the laser and then estimates the distance information of the target. The lateral resolution of the system is 200 × 200 pixels. In 2018, Chen Zhen and Bo Liu built a laser 3D imaging system using the fundamental principle of EO modulation of KDP crystals [9,10,11]. The system uses EMCCD as the imaging detector; the lateral resolution of EMCCD is 1024 × 1024 pixels, and the modulation shutter width of the KDP crystal is 0.32 μs. When using the 3D imaging system to image a target at a distance of 960 m, the range resolution of the system is about 1 m. In 2020, Dr. Song undertook research regarding laser 3D imaging at a close distance and large field of view [16]. Song used a KTN crystal for EO modulation to image the target at a distance of 15 m, with a distance error of 4.8 cm, a distance accuracy of 4.4 cm, and an imaging field of view of 20°. Nevertheless, the growth of KTN crystals is unsatisfactory, and the aperture of the crystal is only 5 mm, which will waste a considerable amount of target echo information and lower the imaging quality.

In the past two years, our research group has conducted preliminary research and exploration of the three-dimensional imaging of EO crystal modulation [17,18,19]. We analyzed and considered the modulation characteristics of EO crystals and built a three-dimensional imaging system for EO crystal modulation. Given the phase difference of EO crystal modulation, a correction algorithm is proposed to improve the imaging precision of the imaging system.

Imaging simulation is an important method to study the theory of laser 3D imaging. Xiao introduced a laser 3D imaging simulation method based on the target model [20], which used CAD software (AutoCAD 2007)to establish the target 3D model and generated the 3D image of pulsed lidar through simulation. In view of the inapplicability of the current ICCD model in the 3D imaging system, Pan improved the noise model in the ICCD model [21], adopted the nonlinear gain model, carried out the time discretization of the model, and carried out systematic simulation and quantitative analysis by combining the illumination model and the 3D data of the object. Dr. An combined the compressive sensing theory with the electro–optical crystal-modulated three-dimensional imaging method [22] and successfully recovered the three-dimensional information of the target by imaging the target at a distance of 100 m through the simulation method. Zhang carried out research on flash 3D imaging lidar [13] and simulated and compared the imaging results of spectroscopic imaging and sub-focal plane imaging. Previous scholars mainly used the research on laser 3D imaging simulation to verify the correctness of the imaging theory and paid little attention to the target scattering characteristics during imaging. Since the distance information recovered by laser 3D imaging is related to the scattering intensity of the target, the target laser scattering model can be better fitted in the process of laser imaging. In this paper, we focus on the establishment of the laser scattering model of the target scene, and the parameters of the target laser scattering characteristic model are fitted through the optimization algorithm to achieve high-precision simulation imaging of the three-dimensional target.

## 2. Principles of 3D Imaging Based on EO Crystal Modulation

### 2.1. Imaging Principles

**Figure 1:**
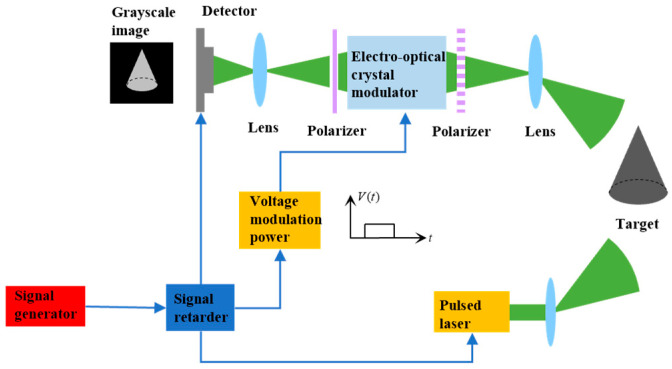
Principle diagram of 3D imaging based on EO crystal modulation.

Three-dimensional imaging based on EO crystal modulation is an active imaging technology that provides active illumination and uses the camera to detect the scattered echo of the target and recover the target distance information. The imaging system mainly comprises a high-power pulsed laser, an emitting optical system, a receiving optical system, a high-performance imaging detector, a signal synchronization control system, an EO crystal modulation system, and an image processing system. The principle of 3D imaging based on EO crystal modulation is shown in Figure 1. In Figure 1, the signal generator emits a reference signal, and the signal retarder receives the reference signal and sends out three delay signals, respectively activating the laser, the EO crystal modulator, and the imaging detector. The laser acquires a trigger signal and emits a pulsed laser to irradiate the target. The laser echo bearing the target information is transmitted to the EO crystal, and the delay signal activates the EO crystal modulator, opens the crystal switch, and realizes the gating function of the target imaging information using the electro–optical crystal modulation. Meanwhile, to guarantee that the target information can be obtained, the detector is set in the external trigger working mode, and the third signal of the signal retarder initiates the detector to open the global shutter and acquire the target imaging gray map. At last, the distance depth map of the target was retrieved based on the target imaging grayscale map, and the laser 3D imaging of the target was completed.

Within the three-dimensional imaging system, the EO crystal offers the detector a high repetition rate and a narrow laser pulse width gating function. The modulation of the EO crystal affects the quality of laser 3D imaging, which is one of the key technologies of three-dimensional imaging. The EO-modulated crystal is a biaxial crystal with a birefringence phenomenon. Assuming that the angle of incidence of the incident rays is α and the azimuth angle is β, when the EO crystal is modulated, one incident ray will produce two refracted rays due to the birefringence effect of the crystal. Assuming that the phase difference of two refracted rays is θ(α,β) and the angular difference in the polarization direction is ϕ(α,β), then the modulated light intensity of the EO crystal can be calculated [17,18]:

(1) $$I(\alpha ,\beta )=\sin^{2}[2\phi (\alpha ,\beta )]\sin^{2}[\frac{\theta (\alpha ,\beta )}{2}]$$

**Figure 2:**
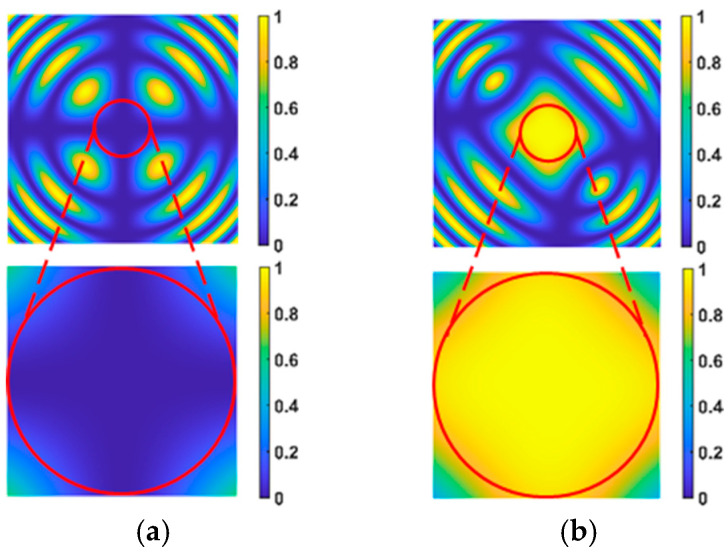
The light intensity distribution of the EO crystal modulation [18].

In accordance with Equation (1), the light intensity distribution of the EO crystal modulation is determined, and the modulation result is demonstrated in Figure 2 below.

In Figure 2, Figure 2a shows the light intensity distribution in the “off” state of the EO crystal, and the bottom half of Figure 2a shows the magnification of the central zone of the upper section. Figure 2b shows the light intensity distribution in the “on” state of the EO crystal, and the lower part of Figure 2b shows the magnification of the central zone of the upper section. Therefore, high-precision gated imaging can be realized by tightly controlling the timing signal and controlling the switching time of EO crystal modulation.

### 2.2. The Principle of Distance Information Solving

**Figure 3:**
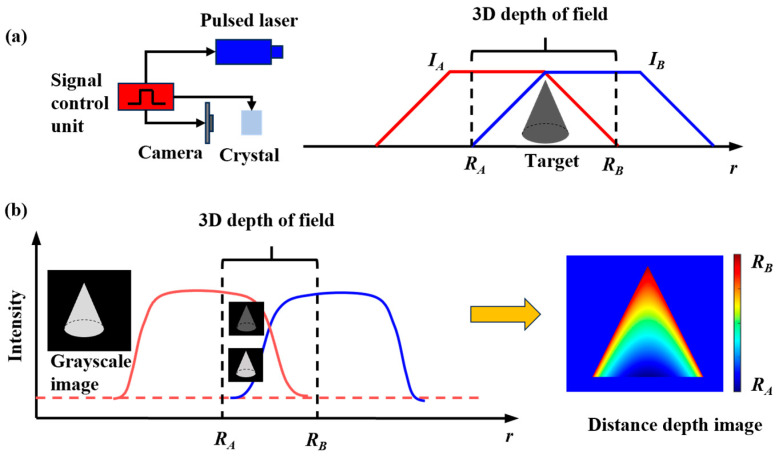
Schematic diagram of EO crystal-modulated 3D imaging range information recovery.

Three-dimensional imaging based on EO crystal modulation can calculate the distance data of the target through the functional relationship between the laser echo energy and the distance information. Trapezoidal distance energy solving is a classical and effective method for solving distance information. The principle of the trapezoidal method can be characterized as the function of the target echo energy acquired by laser 3D imaging, and the target’s distance is approximately trapezoidal. The target energy is exhibited in the form of a gray value in the image, so the distance information of the target can be resolved via the gray value of the image. Figure 3 shows an EO crystal-modulated 3D imaging range information recovery schematic diagram. The camera acquired two raw grayscale images *I_A_* and *I_B_*, with trapezoidal range intensity profiles. In Figure 3b, the distance scope *R_A_*-*R_B_* of the target is recovered from the two grayscale images of the target obtained.

In Figure 3, during an imaging cycle, the energy of the echo signal scattered by the target and detected by the laser 3D imaging detector can be calculated as:

(2) $$R_{N}\left(z\right)=P_{\det}\left(t\right)*G_{N}\left(t\right)=\int_{0}^{+\propto }P_{\det}\left(t\right)\cdot G_{N}\left(t-\frac{2z}{c}\right)dt$$

$R_{N}\left(z\right)$ represents the target energy at *z* within the detector detection range; $P_{\det}\left(t\right)$ denotes the laser pulse energy, *c* stands for the speed of light, and $G_{N}\left(t\right)$ is the waveform expression of the EO modulation crystal gate. When the gate is opened, $G_{N}\left(t\right)=1$ and at other times is 0. When the target distance *z* varies, the received echo energy $R_{N}\left(z\right)$ changes correspondingly so that the relationship between the echo energy $R_{N}\left(z\right)$ and the target distance *z* is set up, which is named the 3D imaging distance energy relationship.

The red and blue curves in Figure 3 are range-energy correlation curves, and the laser echo energy value is not a unique correspondence with the target distance z, so it is essential to utilize multiple distance slice images to address the issue that the above image intensity values cannot be paired one-to-one with the distance. The gray scale of the target in the range-gated image is influenced by the distance between the target and the imaging system, the reflectivity of the target itself, and other elements. Consequently, it remains a problem to directly utilize the image’s gray value to calculate the target’s distance information. To preclude the influence of the aforesaid factors, the relationship between the echo energy received by the distance gating gate and the target distance *z*, that is, the relationship between the pixel gray value of the distance gating image and the target distance *z*, requires being normalized: the function $I_{N}\left(z\right)$ is defined for the target point at the distance *z*, the ratio of the actual echo energy gained under specific lighting conditions to the maximum echo energy that can be received under ideal lighting conditions, so that the normalization of the echo signal energy can be attained, and $I_{N}\left(z\right)$ can be calculated as:

(3) $$I_{N}\left(z\right)=\frac{Q_{N}\left(t\right)}{\max\left(Q_{N}\right)}=\frac{L}{L_{\max}}$$

where $Q_{N}\left(t\right)$ represents the actual echo energy received, $\max\left(Q_{N}\right)$ represents the maximum echo energy that can be received, *L* is the pixel gray value of the target in the slice image at the distance *z*, $L_{\max}$ is the maximum gray value of the distance slice image, and the maximum value of the $I_{N}\left(z\right)$ function is 1.

**Figure 4:**
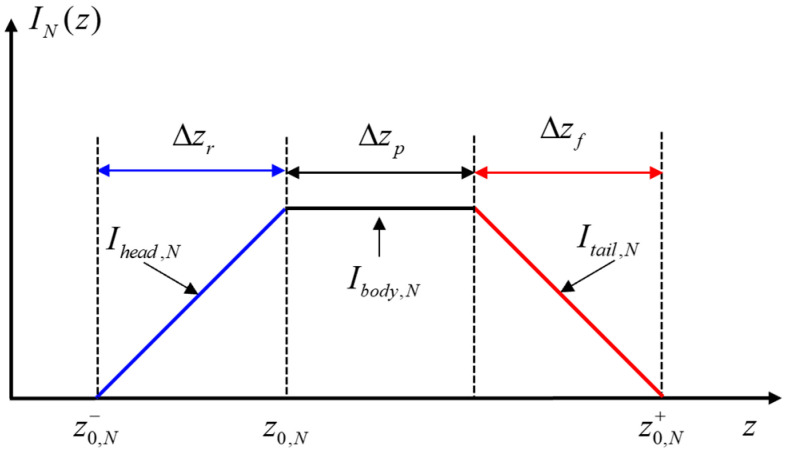
Distance grayscale curve of a trapezoid.

The waveform of the laser pulse width and shutter response jointly determine the shape of the distance grayscale curve. Figure 4 shows the form of the distance grayscale curve of the trapezoid.

In Figure 4, $z_{0,N}^{-}$ and $z_{0,N}^{+}$, respectively, denote the minimum and maximum distance information included in the distance slice image. The $I_{N}\left(z\right)$ functions in the corresponding sub-intervals of $\Delta z_{r}$, $\Delta z_{p}$, and $\Delta z_{f}$ are called “head” signals, “body” signals, and “tail” signals, which are denoted as $I_{head,N}$, $I_{body,N}$, and $I_{tail,N}$, respectively. The values of $\Delta z_{r}$, $\Delta z_{p}$, and $\Delta z_{f}$ are the depth of field of the corresponding sub-section.

Calculate any distance *r* within the effective distance interval, and the computation formula is as follows:

(4) $$r=\left\{\begin{array}{ll} z_{0,N}+\frac{I_{head,N+1}\left(z\right)}{I_{body,N}}\cdot \frac{c\cdot \tau_{p}}{2} & ,z\in \left[z_{0,N},z_{0,N}+c\cdot \tau_{p}/2\right]\\ z_{0,N+1}+\left[1-\frac{I_{tail,N}\left(z\right)}{I_{body,N+1}}\right]\cdot \frac{c\cdot \tau_{p}}{2} & ,z\in \left(z_{0,N}+c\cdot \tau_{p}/2,z_{0,N}+c\cdot \tau_{p}\right] \end{array}\right.$$

After the target’s gray image is acquired using the crystal polarization modulation range gating imaging system, the distance of the target is restored by applying the above calculation formula, and the distance depth map of the target is acquired.

## 3. Study the Laser Scattering Characteristics of the Target

According to the three-dimensional imaging principle of EO crystal modulation, the intensity information of the echo obtained by the detector is a function of the target distance. Therefore, it is of great significance to study the laser scattering characteristics of the target and construct the model of the laser scattering characteristics of the target to improve the accuracy of 3D imaging. According to the laser scattering characteristics of the target, the model can calculate the reflected light intensity of the target at any reflection angle and azimuth angle. The research idea of this paper is to establish a model of the laser scattering characteristics of the target. Secondly, an experimental device was set up to measure the scattered light intensity of the target material at different angles. Then, according to the measurement results, an optimal fitting algorithm was proposed to calculate the model parameters. Finally, the solved model is used to analyze the laser scattering characteristics of the target.

### 3.1. Five-Parameter BRDF Model

**Figure 5:**
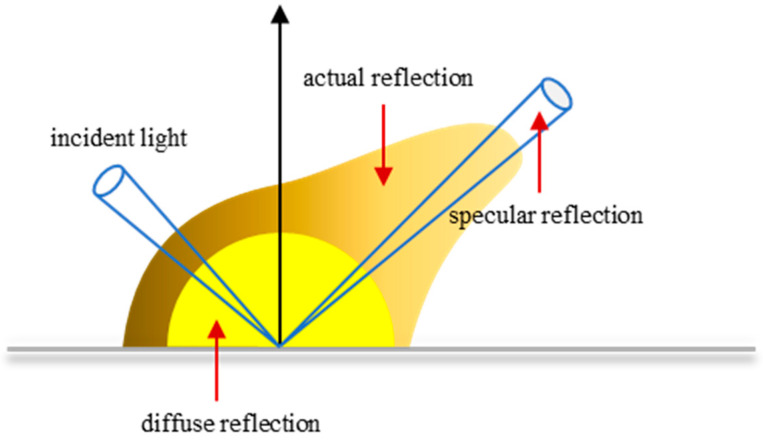
Schematic diagram of the reflection on the surface of an object.

The reflection of the surface of the target material can be divided into diffuse reflection and specular reflection, as shown in Figure 5. Diffuse reflection means that the target material reflects the incident light uniformly in all directions, and the light intensity in the target reflection space is constant; the diffuse reflection target can be called a Lambert body. When the surface of the target material is relatively smooth, the energy reflected by the target is concentrated in one direction, and the reflection angle is equal to the incident angle, which is called specular reflection. However, in practice, the reflection of the surface of the object often has two forms of specular reflection and diffuse reflection at the same time, and there are uneven reflections of the target in all directions.

**Figure 6:**
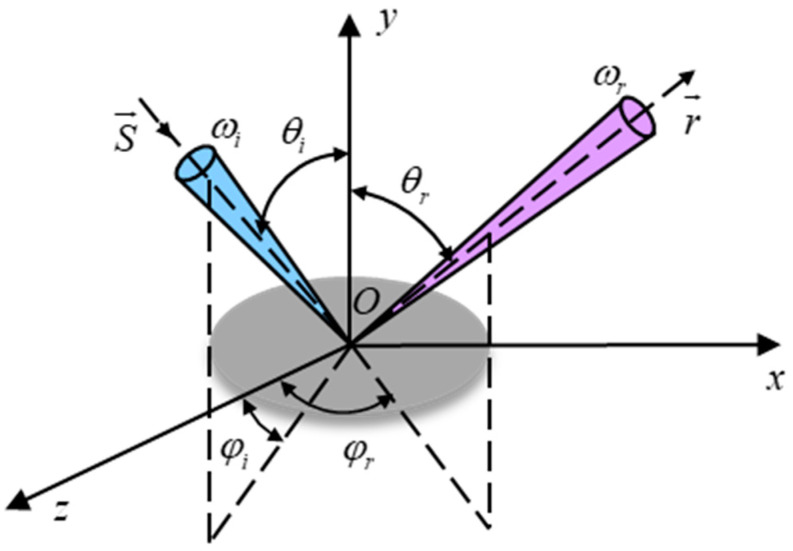
The geometric relationship of the BRDF.

In 1970, the scholar Nicodemus proposed the bidirectional reflection distribution function concept. BRDF models were divided into the analytical model [23,24,25,26,27] and the empirical model [28,29,30,31,32] according to different modeling methods. The physical meaning of BRDF is the ratio of the micro-increment from the directional irradiance and the increment in the reflected radiant brightness in the direction it induces in sr^−1^. In Figure 6, a light source evenly illuminates the target’s surface, and the face element dA’s irradiance in the $s(\theta_{i},\phi_{i})$ order of the solid angle element $d\omega_{i}$ is $dE_{i}(\theta_{i},\phi_{i})$. The radiance of the face element dA in the solid angle $d\omega_{r}$ along the $r(\theta_{r},\phi_{r})$ after irradiation is $dL_{r}(\theta_{i},\phi_{i};\theta_{r},\phi_{r})$, then the definition of BRDF is as follows:

(5) $$BRDF\left(\theta_{i},\varphi_{i},\theta_{r},\varphi_{r}\right)=\frac{dL_{r}(\theta_{i},\varphi_{i};\theta_{r},\varphi_{r})}{dE_{i}(\theta_{i},\varphi_{i})}$$

The unit of radiant brightness is W/(m^2^·sr), which is defined as the radiant flux per unit area and solid angle along the radiation direction:

(6) $$L_{r}(\theta_{i},\varphi_{i};\theta_{r},\varphi_{r})=\frac{d\Phi_{r}(\theta_{i},\varphi_{i};\theta_{r},\varphi_{r})}{dA\cos\theta_{r}d\omega_{r}}$$

The unit of irradiance is W/m^2^ and is defined as the radiant flux received per unit area as shown below:

(7) $$E_{i}(\theta_{i},\varphi_{i})=\frac{d\Phi_{i}(\theta_{i},\varphi_{i})}{dA}$$

Generally, the BRDF of different types of material surfaces is diverse and complex. In reflectance measurement experiments, the BRDF of various materials can be measured and evaluated using a laser power meter and a standard plate, and the scattering characteristics of the material can be analyzed based on the actual measurement results.

**Figure 7:**
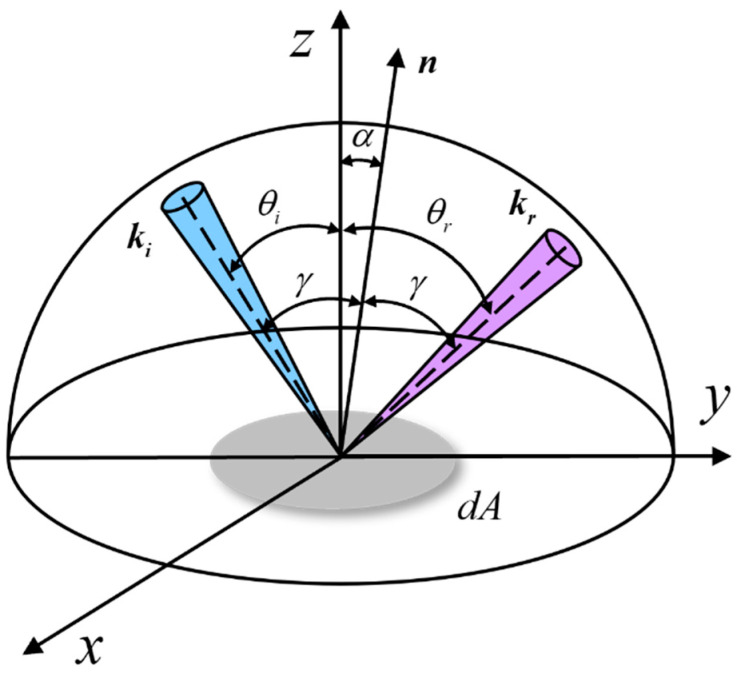
Five-parameter BRDF.

This paper researches the reflection characteristics of aluminum plates and gold foil in the five-parameter BRDF model. This model considers the material surface to be composed of many small surface elements, and the reflection of each surface element follows Fresnel’s reflection law. Figure 7 presents a diagram of the BRDF model of the microelement. The oz axis is the average direction of the macroscopic object surface, and the n axis is the average direction of the microfacet. γ is the angle between the incident light’s leadership and the microface’s usual direction. The incident wave vector $k_{i}$ illuminates the material’s surface, and $k_{r}$ is the reflected wave vector. $\left(\theta_{i},\varphi_{i}\right)$ is the incident angle, $\left(\theta_{r},\varphi_{r}\right)$ is the scattering angle, α is the included angle between the n axis and z axis, α and γ can be represented as:

(8) $$\cos\alpha =\frac{\cos\theta_{i}+\cos\theta_{r}}{2\cos\gamma }$$

(9) $$\cos^{2}\gamma =\frac{1}{2}\left(\cos\theta_{i}\cos\theta_{r}+\sin\theta_{i}\sin\theta_{r}\cos\varphi_{r}+1\right)$$

According to the principle of geometric optics and probability, the five-parameter model can be expressed as:

(10) $$\frac{f_{r}\left(\theta_{i},\theta_{r}\right)}{\cos\theta_{i}}=k_{b}\frac{k_{r}^{2}\cos\alpha }{1+\left(k_{r}^{2}-1\right)\cos\alpha }\exp\left[b\cdot {\left(1-\cos\gamma \right)}^{a}\right]\cdot \frac{G\left(\theta_{i},\theta_{r}\right)}{\cos\theta_{i}\cos\theta_{r}}+\frac{k_{d}}{\cos\theta_{i}}$$

where $k_{b}$, $k_{d}$, $k_{r}$, a, and b are unknown parameters. $k_{b}$ and $k_{d}$ are specular reflection coefficient and diffuse reflection coefficient, respectively. $k_{r}$ is related to the roughness of the material surface. a and b depend on the refractive index of the material. $f_{r}\left(\theta_{i},\theta_{r}\right)$ is the BRDF measured value, and $G\left(\theta_{i},\theta_{r}\right)$ is the shadow shading function of the BRDF model, which can be expressed as:

(11) $$G\left(\theta_{i},\theta_{r}\right)=\frac{1+\frac{\omega_{p}\left|\tan\theta_{ip}\tan\theta_{rp}\right|}{1+\sigma_{r}\tan\beta_{p}}}{\left[\left(1+\omega_{p}\tan^{2}\theta_{ip}\right)\left(1+\omega_{p}\tan^{2}\theta_{rp}\right)\right]}$$

(12) $$\left\{\begin{array}{c} \omega_{p}=\sigma_{p}\left(1+\frac{u_{p}\sin\alpha }{\sin\alpha +v_{p}\cos\alpha }\right)\\ \tan\theta_{ip}=\tan\theta_{i}\frac{\sin\theta_{i}+\sin\theta_{r}\cos\varphi_{r}}{2\sin\alpha \cos\beta }\\ \tan\theta_{rp}=\tan\theta_{r}\frac{\sin\theta_{r}+\sin\theta_{i}\cos\varphi_{r}}{2\sin\alpha \cos\beta }\\ \tan\beta_{p}=\frac{\left|\cos\theta_{i}-\cos\beta \right|}{2\sin\alpha \cos\beta } \end{array}\right.$$

where $\sigma_{p}$, $\sigma_{r}$, $u_{p}$, and $v_{p}$ are the experimental parameters related to the material’s surface roughness and refractive index. Generally, we set the following empirical values to $\sigma_{p}$ = 0.0136, $\sigma_{r}$ = 0.0136, $u_{p}$ = 9.0, $v_{p}$ = 1.0.

### 3.2. Experimental Equipment and Methods

**Figure 8:**
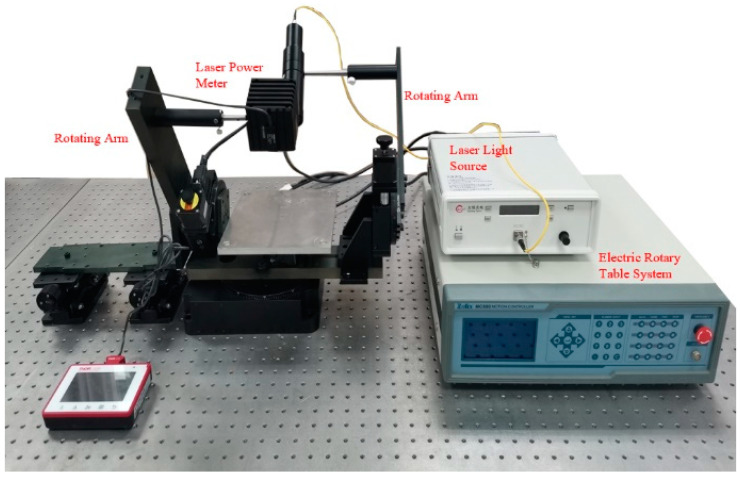
BRDF measurement system.

According to Equation (5), the measured value of BRDF can be expressed as the ratio of the irradiance reflected by the target to the irradiance irradiated on the surface of the target. To obtain the actual data of the target material, we independently designed a set of multi-azimuth irradiance measurement systems to research the BRDF characteristics of the aluminum plate and gold foil. Figure 8 presents a diagram of the measurement system. The measuring system comprises a laser light source, an electric rotary table system, and a high-sensitivity laser power meter. Using a rotating arm for the laser light source, another for the laser power meter, and two corresponding independent rotary motors makes material surface measurement possible. Adjust the height of the lifting platform so that the axis of the two rotating arms is the same height as the material’s surface to be measured. The system can complete high-precision measurements under the setting of an electric rotary table.

In the actual measurement of BRDF, factors such as experimental environment and measuring equipment may cause changes in measured values, called experimental uncertainty. We assume that experimental uncertainty is mainly related to the laboratory environment, detector, laser light source, and rotation device. The measurement uncertainty model of the BRDF measuring system can be expressed as:

(13) $$U_{BRDF}=\sqrt{{U_{E}}^{2}+{U_{D}}^{2}+{U_{L}}^{2}+{U_{T}}^{2}}$$

The experiment was conducted in an opaque dark room, and a laser light source (λ = 650 nm) supplied stable collimated illumination. A 5 h test indicated that the output power error of the laser light source was within 3%. The high-precision power meter used in the experiment is Thorlabs S120-FC, and the product test report shows that the measurement uncertainty is within 3% under the condition of wavelength of 440–980 nm. The experimental measurement error was 0.03 μW/m^2^ of the irradiance under dark shapes in the laboratory; the uncertainty introduced by the laboratory environment is about 0.15%. The accuracy of the incidence angle and reflection angle is mainly affected by the rotation arms, and the uncertainty of the rotation arm is 0.3%. Therefore, according to the calculation in Formula (13), the fate of the BRDF measuring system we designed is 3.6%. It should be noted that the experiment ignored the uncertainty caused by non-ideal factors such as operating error and the laboratory temperature.

**Figure 9:**
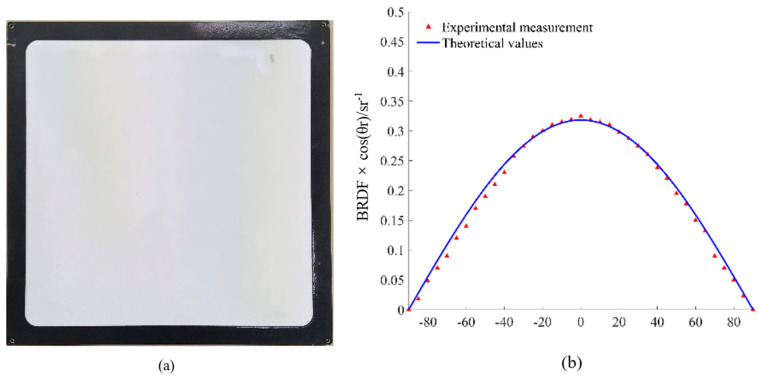
(**a**) Lambertian plate. (**b**) Experimental measurement and theoretical values.

This paper measured the BRDF of the Lambertian plate shown in Figure 9a to verify our measuring system further before measuring the BRDF of the target material. Zenith angle and azimuth angle are set as $\theta_{i}=\varphi_{i}=0{}^{\circ}$ due to the isotropy of the Lambertian plate, and the reflection angle ranges from 0° to 90° with an interval of 5°. Experimental measurement and theoretical values are shown in Figure 9b.

The experimental measurement is in good agreement with the theoretical values. The reflection error of the Lambertian plate is less than 0.53%, which indicates that our practical device is accurate and reliable.

The aluminum sheet and gold leaf samples are placed on a horizontal platform. The incidence angle of the laser light source is changed by controlling the angle of the light source arm, and the incidence angles were 15°, 30°, 45°, and 60°, respectively. For each inclination of incidence, the detector automatically rotates from −90° to 90° to collect the reflected beam in the plane. The experimental test results will be used to optimize the BRDF model parameters.

## 4. Hybrid BBO–Firefly Optimization Algorithm

When solving practical problems, intelligent algorithms are often needed to build suitable models for optimal solving [33,34,35]. Therefore, after obtaining the BRDF data of the target material through a self-designed experimental setup, the unknown parameters of the BRDF model need to be fitted by the algorithm. To accurately describe the characteristics of the target material, the hybrid BBO–Firefly algorithm is proposed to solve the parameter optimization problem of the BRDF model. The hybrid BBO–Firefly algorithm is a new joint optimization algorithm combining the BBO and Firefly algorithms. It not only guarantees the global search characteristic of the BBO algorithm but also acts as the fast search characteristic of the Firefly algorithm.

### 4.1. BBO Algorithm

**Figure 10:**
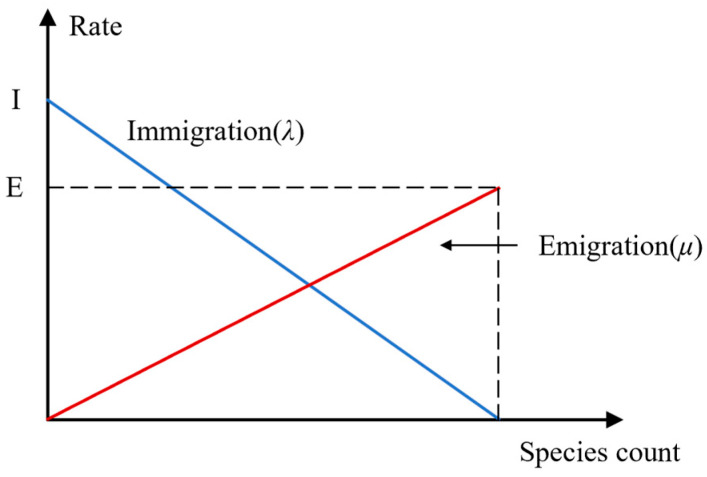
Species model of a habitat.

The biogeography-based optimization (BBO) algorithm is based on the biogeography theory proposed by Dan Simon in 2009 [36]. The algorithm simulates the migration and variation process in biogeography to evolve the population and solve the optimization problem continuously. The algorithm could be understood as the survival of organisms on islands isolated from other habitats. Affected by external conditions such as rainfall and ambient temperature, some microorganisms in the population will migrate to better adapt to the environment. The habitat quality of a habitat is expressed by the habitat suitability index (HIS), and a high HSI means that the habitat is geographically well suited to the species’ survival. Factors related to HSI include rainfall, vegetation diversity, geomorphological characteristics, land area, temperature, humidity, etc., called suitability index variables (SIV). When high HSI habitats or low HSI habitats have more species, the species may migrate to adjacent habitats. This process is called emigration. During immigration, species move towards the high HSI habitat with few species. The immigration and emigration of species in a habitat are known as migration. The migration of species in their habitats is shown in Figure 10.

In Figure 10, when there are no species in a habitat, the immigration rate λ of the habitat will attain its maximum I. In addition, as the number of species continues to increase, only a few species can survive successfully. Thus, the immigration rate reduces and turns to 0 at S = Smax. Likewise, taking into account the emigration curve in Figure 10, the migration rate μ increases with increasing species, so habitats with more crowded species have higher emigration rates than habitats with fewer populations.

The BBO algorithm is employed to solve optimization issues, and an optimization problem can be described as:

(14) $$\begin{array}{cc} \;\mathrm{Max}\; & f\left(x\right)\\ \;s.t.\; & g\left(x\right)\leq 0\\ & x_{i}^{\min}\leq x_{i}\leq x_{i}^{\max}\;\;\;\;i=1,2,\ldots ,n \end{array}$$

where f(x) indicates the objective function, $g\;\left(x\right)$ indicates the set of all constraints, and $x=(x_{1},x_{2},\ldots ,x_{n})$ is the decision variable. $x_{i}^{\min}$ and $x_{i}^{\max}$ are the minimum and maximum values of variable $x_{i}$ s, respectively. Solving an optimization problem could be described as finding the total value of the objective function f(x) under the constraint $g\;\left(x\right)$. In the initialization phase of the BBO algorithm, we need to set its parameters. The parameters include the maximum species count $S_{\max}$, migration rate E, immigration rate I, the maximum mutation rate $m_{\max}$, the number of habitats, and the number of iterations.

In the BBO algorithm, each individual or decision variable is considered a habitat with a HSI. The initial values of these habitats are randomly initialized as $x_{i}^{\min}+U_{d}(x_{i}^{\max}-x_{i}^{\min})$. $U_{d}\in (0,1)$ is the uniformly distributed random number. An N-dimensional vector represents each habitat as $H=[SIV_{1},SIV_{2},\ldots ,SIV_{N}]$. HSI is determined by finding the objective function of *H*, i.e., HSI=f(H).

The optimization process of the BBO algorithm consists of two parts: migration and mutation. Migration is an adaptation process to update the SIV value of existing habitats, including emigration and immigration. The algorithm evolves the solution to the optimization problem by changing the migration rate λ and the migration rate μ. Another habitat is selected based on the emigration rate μ, and its SIV will be randomly migrated to the SIV of the chosen habitat. Initially, the rate of emigration and the rate of migration were a function of the number of species in the habitat and were assessed as:

(15) $$\lambda_{k}=I\left(1-\frac{k}{S_{\max}}\right)\;\;\;\;;\;\;\;\;\mu_{k}=E\left(\frac{k}{S_{\max}}\right)$$

where *I* and *E* are the maximum possible immigration rate and emigration rate, respectively. *k* is the number of the species of the kth individual. $S_{\max}$ is the maximum number of species.

In addition to migration, in BBO, mutations are used to increase the diversity of the population so that the best solution can be obtained. It is assumed that there is a total of $S_{\max}$ habitats and that there are *S* species in the $S_{th}$ habitat. For the $S_{th}$ habitat, the immigration rate is $\lambda_{s}$ and emigration rate is $\mu_{s}$. $P_{S}$ is the probability that there are exactly *S* species in the habitat, then $P_{S}$ changes from time t to t+Δt, given as:

(16) $$P_{S}\left(t+\Delta t\right)=P_{S}\left(t\right)\left(1-\lambda_{s}\Delta t-\mu_{s}\Delta t\right)+P_{S-1}\left(t\right)\lambda_{s-1}\Delta t+P_{S+1}\left(t\right)\mu_{k+1}\Delta t$$

When t → 0, Equation (12) can be expressed as:

(17) $${\dot{P}}_{S}=\left\{\begin{array}{l} -\left(\lambda_{s}+\mu_{s}\right)P_{s}+\mu_{S+1}P_{S+1}\;\;\;\;S=0\\ -\left(\lambda_{s}+\mu_{s}\right)P_{s}+\mu_{S-1}P_{S-1}+\mu_{S+1}P_{S+1}\;\;\;\;1\leq S\leq S_{\max}-1\\ -\left(\lambda_{s}+\mu_{s}\right)P_{s}+\mu_{S-1}P_{S-1}\;\;\;\;\;S=S_{\max} \end{array}\right.$$

where $P_{S-1}$, $P_{S}$, and $P_{S+1}$ are the species count probability; $\lambda_{s-1}$, $\lambda_{s}$, and $\lambda_{s+1}$ are the immigration rate; and $\mu_{s-1}$, $\mu_{s}$, and $\mu_{s+1}$ are the emigration rate of the habitat with *S* − 1, *S*, and *S* + 1 species, respectively. $S_{\max}$ is the maximum species count in the habitat. The steady-state probability of the quantity of each species is given below:

(18) $$P_{0}={\left[1+\sum_{j=1}^{S_{\max}}\prod_{i=1}^{l}\frac{\lambda_{i-1}}{\mu_{i}}\right]}^{-1}$$

(19) $$\begin{array}{cc} P_{k} & =P_{0}\prod_{i=1}^{k}\left(\frac{\lambda_{i-1}}{\mu_{i}}\right)\\ & =\frac{1}{1+\sum_{j=1}^{S_{\max}}\prod_{i=j}^{l}\frac{\mu_{i}}{\lambda_{i-1}}+\sum_{l=k+1}^{S_{\max}}\prod_{i=k+1}^{l}\frac{\lambda_{i-1}}{\mu_{i}}},k\in \left\{1,2,\ldots ,S_{\max}\right\} \end{array}$$

When *E* = *I*, based on the probability of the randomly selected SIV habitat, the mutation rate *m* can be expressed as:

(20) $$m_{i}=m_{\max}\times \left(1-\frac{P_{i}}{p_{\max}}\right)$$

where $m_{\max}\in [0,1]$ is called maximum mutation probability. P_max_ = max(P_1_, P_2_,…, P_NP_), *NP* is the number of habitats, and $P_{i}$ is the probability of habitat *i*, which can be calculated by (18) and (19). The pseudo-code of the BBO algorithm is depicted in Algorithm 1.
**Algorithm 1: Biogeography-Based Algorithm**1:  **// BBO parameter initialization //**2:  Create a random set of habitats (population) $H=[SIV_{1},SIV_{2},\ldots ,SIV_{N}]$;3:  Compute corresponding HSI values HSI=f(H);4:  **While**
5:       Compute immigration rate *λ* and emigration rate *μ* for each habitat based on HSI;6:       **// Migration //**7:       Select $H_{i}$ with probability based on $\lambda_{i}$;8:       **If** $H_{i}$ is selected9:            Select $H_{j}$ with probability based on $\mu_{j}$;10:            **If** $H_{j}$ is selected11:                 Randomly select an SIV from $H_{j}$;12:                 Replace a random SIV in $H_{i}$ with one from $H_{j}$;13:           **End if**14:      **End if**15:      **// Mutation//**16:      Select an SIV in $H_{i}$ with probability based on the mutation rate;17:      **If** $H_{i}$ (SIV) is selected18:           Replace $H_{i}$ (SIV) with a randomly generated SIV;19:      **End if**20:      Recompute HSI values;21:  **End while**22:  Show the results.

### 4.2. Firefly Algorithm

The Firefly algorithm is a natural heuristic optimization algorithm proposed by Yang in 2010 [37]. The algorithm simulates the luminescence mechanism and behavior mode of tropical fireflies in nature. It stimulates the search and position updating process in the optimization process into the mutual attraction and movement process among fireflies. The objective function is simulated as the luminance information of an individual firefly, and the optimal solution is found by iteration.

In the Firefly algorithm, the attraction between fireflies is determined by brightness, which is proportional to the value of the target fitness function. Thus, the relationship between the intelligence *I (X)* of the Firefly *X* and its objective function *f* (*X*) can be expressed as *I* (*X*) *∝ f* (*X*).

The attraction *β* between two fireflies depends on their distance *r*. As the distance increases, the attraction gradually decreases. Suppose there are *M* fireflies: X_1_, X_2_, …, X_M_. The attraction $\beta (r_{ij})$ between the two fireflies $X_{i}$$X_{j}$ could be expressed as:

(21) $$\beta \left(r_{ij}\right)=\beta_{0}e^{-\gamma r_{ij}^{2}}$$

(22) $$r_{ij}=∥X_{i}-X_{j}∥=\sqrt{\sum_{d=1}^{D}{\left(x_{id}-x_{jd}\right)}^{2}}$$

where *D* is the dimension of the problem to be optimized, the distance between $X_{i}$ and $X_{j}$ is $r_{ij}$. $x_{id}$ and $x_{jd}$ are the *d*th component elements of $X_{i}$ and $X_{j}$, respectively. The parameter *β*_0_ is the attraction coefficient. *γ* is called the light absorption coefficient.

Suppose there are two fireflies $X_{i}$, $X_{j}$, and firefly $X_{j}$ is brighter than $X_{i}$, then $X_{i}$ is attracted to $X_{j}$ and moves towards $X_{j}$, and the motion of $X_{i}$ can be expressed as:

(23) $$x_{id}\left(t+1\right)=x_{id}\left(t\right)+\beta_{0}e^{-\gamma r_{ij}^{2}}\left(x_{jd}\left(t\right)-x_{id}\left(t\right)\right)+\alpha \epsilon_{i}$$

where $\epsilon_{i}\in [-0.5,0.5]$ is a random number and α∈[0,1] is the step factor. The pseudo-code of the FA algorithm is depicted in Algorithm 2.
**Algorithm 2: Firefly Algorithm**1:  **// FA parameter initialization //**2:  Create the Population size, *α*, *β_0_*, *γ* and the number of iterations;3:  Calculate the fitness function $f(X_{i})$ of the firefly $X_{i}$, where $X_{i}=(X_{1},X_{2},\ldots ,X_{M})$;4:  **While** (*iter* < Max Generation)5:       **for** *i* = 1:*M* (all *M* fireflies)6:            **for** *j* = 1:*M* (all *M* fireflies)7:            **// Updated the position and brightness//**8:                 **if** $f(X_{i})<f(X_{j})$, move firefly *i* towards *j;*9:                 **End if**10:               Update attractiveness *β* with distance *r*;11:               Evaluate new solution and update $f(X_{i})$ in the same way as (19);12:          **End for** *j*13:     **End for** *i*14:  Rank the solutions and find the best global optimal;15:  **End while**16:  Show the results.

### 4.3. Hybrid BBO–Firefly Algorithm

**Figure 11:**
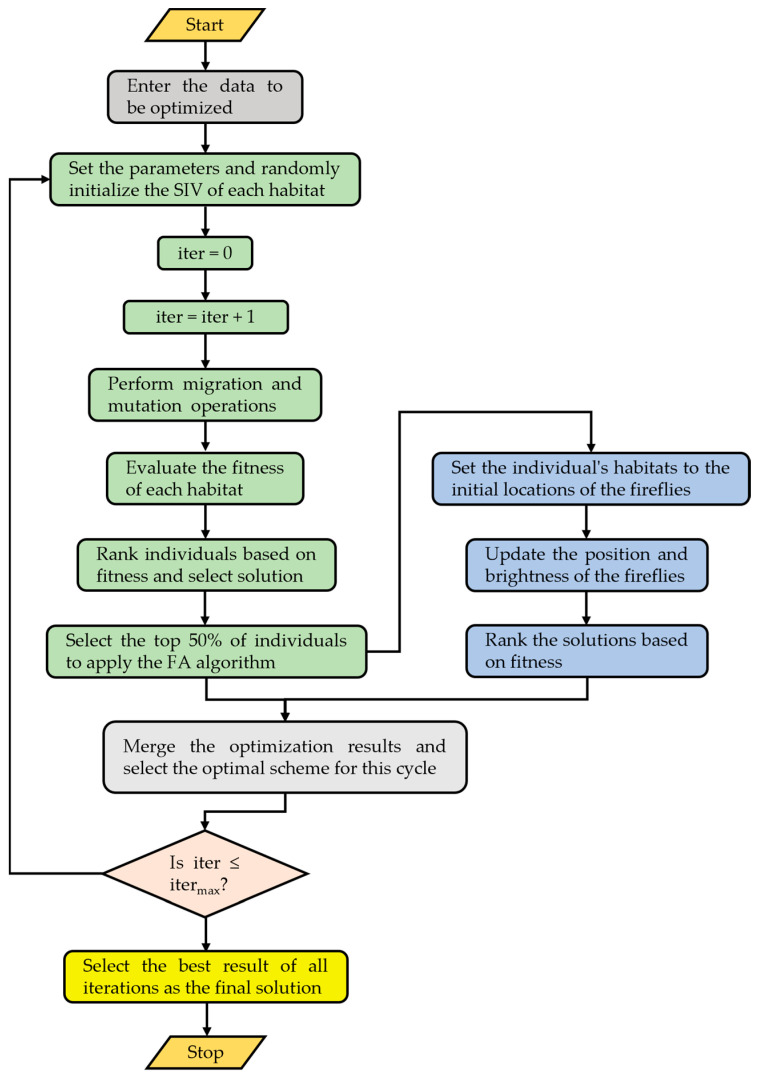
Flowchart of the hybrid BBO–Firefly algorithm.

The hybrid BBO–Firefly algorithm introduces the Firefly operator into the BBO algorithm, improving the local searching ability and solving precision. The basic steps of the hybrid BBO–Firefly can be summarized in the flowchart in Figure 11.

As shown in Figure 11, before optimizing the algorithm, we first need to input the data to be optimized, initializing the algorithm parameters such as m_max_, iter_max_, etc. Next, generate a set of random numbers as the SIV for the habitats. This is followed by a circular computation of the hybrid BBO–Firefly algorithm. In the BBO–Firefly algorithm, firstly, the migration and mutation operations of the BBO algorithm are used to solve the optimization problem. Then, evaluate the fitness of each habitat and rank its results. Then, select the top 50% of individuals to apply the FA algorithm, updating the position and brightness information of fireflies through the FA algorithm. Next, merge the optimization results of the BBO algorithm and the FA algorithm and select the optimal scheme for this cycle. Finally, the best result of all iterations is chosen as the final solution.

According to the flowchart implementation steps in Figure 11, the experimental data were optimized to verify the algorithm’s feasibility.

### 4.4. Experimental Result

**Figure 12:**
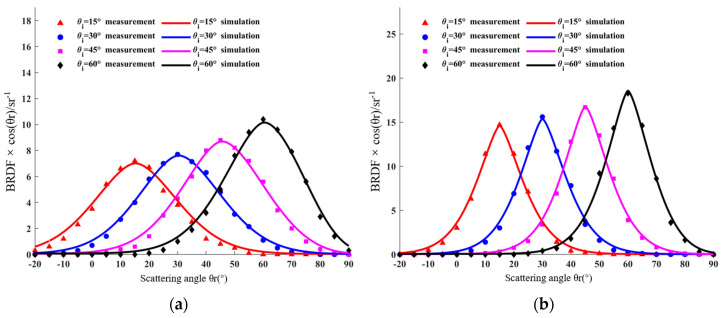
BRDF measurements and fitting curves for aluminum plates and gold foils.

The BRDF measuring device obtained the BRDF data values of aluminum plates, gold foils, and the unknown parameters in the five-parameter BRDF model fitted by the BBO–Firefly algorithm. The BRDF measurements and fitting curves are plotted as shown in Figure 12. The measurements are represented as discrete points, and the fitting function is plotted as curves. Figure 12a,b shows the variation of BRDF measurements and fitted values with observation angles at 15°, 30°, 45°, and 60° incidence conditions for aluminum plates and gold foils, respectively, with the observation angles ranging from −20° to 90°. From the perspective of the algorithm fitting effect, there are differences in the BRDF characteristics of different materials, the peak position of BRDF is different under different incident angles of the same material, and the peak value of BRDF is in the same observation angle direction as the incident angle, which shows that BRDF is composed of diffuse reflection and specular reflection. In the same case, the BRDF value in Figure 12b is greater than in Figure 12a, so the specular reflection of gold foil is stronger than that of the aluminum plate. The fitting curve shows that the model curve calculated by the BBO–Firefly algorithm is in good agreement with the experimental data.

This paper sets up three control experiments to accurately measure the BBO–Firefly algorithm’s optimization effect. In the same situation, the optimization effect of the three groups of algorithms is compared, and the algorithm’s optimization error is evaluated using the MSE function. The objective function is defined as:

(24) $$MSE=\frac{1}{n}\sum_{i=1}^{n}{\left[f_{\mathrm{model}}\left(k_{b},k_{d},k_{r},a,b\right)-f_{\mathrm{measured}\;}\left(k_{b},k_{d},k_{r},a,b\right)\right]}^{2}$$

The BRDF model parameters of the BBO algorithm, Firefly algorithm, and hybrid BBO–Firefly algorithm are calculated, and the MSE function of each algorithm is calculated and written in Table 1.

According to the optimization results in Table 1, the incidence angles are 15°, 30°, 45°, and 60°, respectively. When the material is an aluminum plate, the MSE of the BBO–Firefly algorithm is reduced to 14%, 7%, 10%, and 11% compared with the original BBO algorithm, and the MSE is reduced to 14%, 7%, 12%, and 15% compared with the original Firefly algorithm. When the material is gold foil, the MSE of the BBO–Firefly algorithm is reduced to 17%, 18%, 12%, and 11% compared with the original BBO algorithm, and the MSE is reduced to 15%, 24%, 28%, and 32% compared to the original Firefly algorithm. The results show that the optimization precision of the BBO–Firefly algorithm is much higher than that of the BBO algorithm and Firefly algorithm, and it has better stability performance.

**Figure 13:**
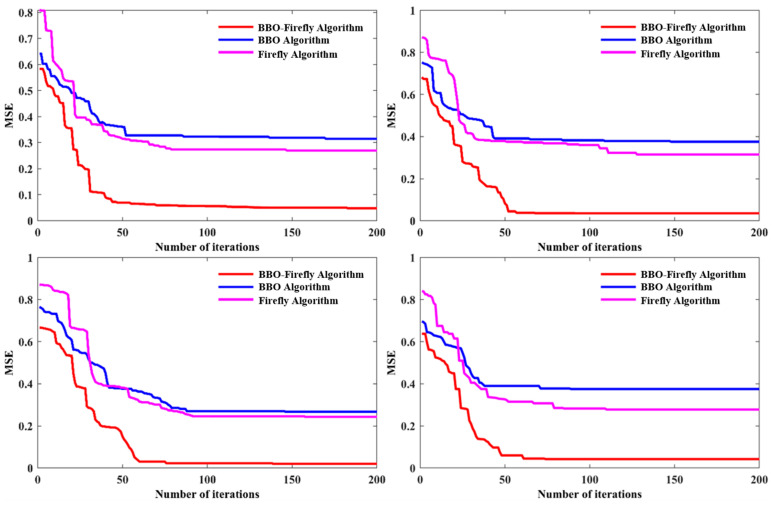
BRDF parameter optimization convergence curve.

For intelligent optimization algorithms, convergence speed is also an important indicator to measure the algorithm’s performance. The BBO–Firefly algorithm, BBO algorithm, and Firefly algorithm were used to optimize the five parameters of BRDF at the incidence angles of 15°, 30°, 45°, and 60°, respectively. The number of iterations was set to 200, and the calculation results are shown in Figure 13. Three lines of different colors represent the results of the calculation fitting of the three algorithms. With the increasing number of iterations, the MSE of the algorithm gradually converges. In Figure 13, the BBO algorithm shows excellent optimization results in the initial iteration, and the Firefly algorithm has better convergence ability. The BBO–Firefly algorithm combines the advantages of both algorithms and tends to be horizontal after 100 iterations. Compared with the BBO algorithm and the Firefly algorithm, the BBO–Firefly algorithm has more vital convergence ability and can quickly search for the optimal solution. By comparing the three algorithms, the BBO–Firefly algorithm shows its superiority in calculation accuracy and iterative efficiency.

**Figure 14:**
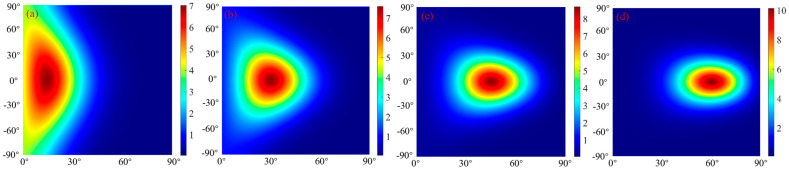

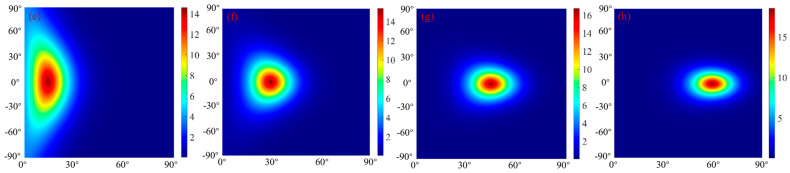
BRDF simulation measurement.

The BBO–Firefly algorithm is used to solve the unknown parameters of the target material’s BRDF model, and the target material and spatial scattering distribution of the target material are established. The spatial distribution of laser scattering of aluminum plates and gold foil is shown in Figure 14.

Figure 14 is a false-color image of the simulated aluminum plate and gold foil BRDF measurements, with the color variation reflecting the scattering distribution of the target in space. Figure 14a–d shows the BRDF distribution of an aluminum plate with incident angles of 15°, 30°, 45°, and 60°, respectively. The peak scattering position of the target is the reflection position corresponding to the incident angle of the laser. The scattering distribution gradually spreads from the peak center, and the intensity gradually decreases. Figure 14e–h shows the BRDF distribution of the gold foil with incidence angles of 15°, 30°, 45°, and 60°, respectively. Comparing the scattering distribution of the two different materials, it can be seen that the specular reflectance of gold foil is more potent than that of the aluminum plate, and there is a significant difference in the reflective characteristics between the two sample materials.

## 5. Three-Dimensional Imaging Simulation Experiments

**Figure 15:**
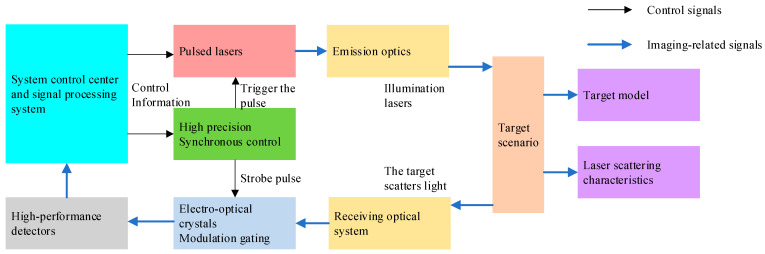
Architecture diagram of laser 3D imaging simulation model.

To verify the principle and numerical analysis of the 3D imaging method based on EO crystal modulation, a laser 3D imaging simulation system is established by using the simulation method. In order to make the simulation effect more consistent with the actual situation, the laser 3D imaging system comprehensively considers the influence of various factors, including the 3D scene of the virtual target, the illumination system model, the receiving system model, atmospheric turbulence, and backscattering. Based on the above factors, the architecture of the laser 3D imaging simulation model is shown in Figure 15.

**Figure 16:**
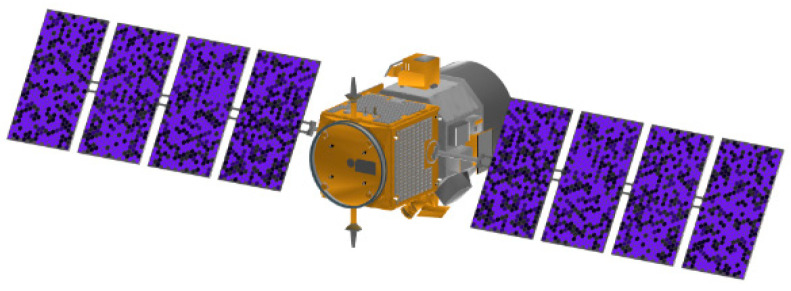
CALIPSO satellite model.

Performing 3D imaging requires the selection of suitable imaging targets. In this paper, the Cloud-Aerosol Lidar and Infrared Pathfinder Satellite Observations (CALIPSO) model was downloaded from the official website of the National Aeronautics and Space Administration (NASA) in the United States as an imaging target. The CALIPSO satellite is a scientific satellite that has recorded more than 10 billion lidar measurements in the course of 17 years of operation, providing important data support for scientists to build more complex atmospheric models. Figure 16 shows the CALIPSO satellite model.

**Figure 17:**
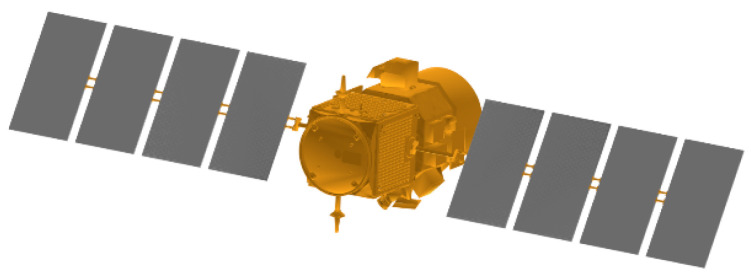
Laser 3D imaging target model.

According to Figure 16, the CALIPSO satellite shell is composed of a variety of materials, each with different color information. In the laser 3D imaging simulation experiment in this paper, in order to facilitate the analysis and calculation, we have formed a reasonable simplified setting for the surface material of the satellite model. In practice, satellites can generally be divided into two parts: the main body of the satellite and the solar sail panels, so we set that the surface material of the satellite’s main body is made of metal gold, and the solar sail panels of the satellite are made of metal aluminum. Figure 17 shows the satellite model for simplified material analysis.

For the 3D imaging simulation of the satellite, the BRDF model of the satellite established in Section 3.1 is used for the imaging experiment, and the actual experimental parameter values are the best parameters fitted by the hybrid BBO–Firefly algorithm proposed in Section 4.3 of the paper. Compared with the existing methods, the 3D imaging simulation method proposed in this paper can scientifically and effectively present and analyze the influence of laser scattering on the imaging results in the process of laser 3D imaging, which can provide a basis for related theoretical research through the closed-loop process of setting up experimental devices, experimental data acquisition, satellite BRDF model construction, model parameter optimization and solving, and simulating satellite laser scattering characteristics by solving model parameters.

**Figure 18:**
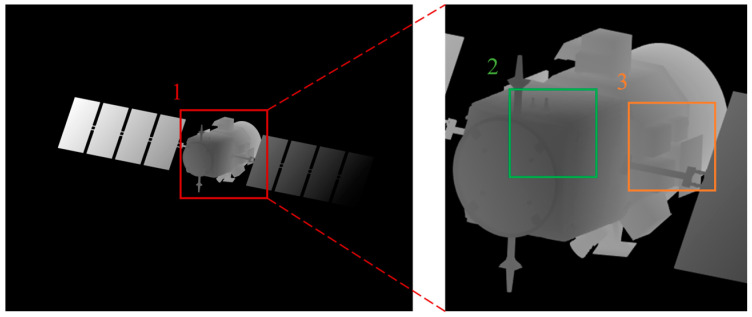
The results of laser 3D imaging (conventional methods).

**Figure 19:**
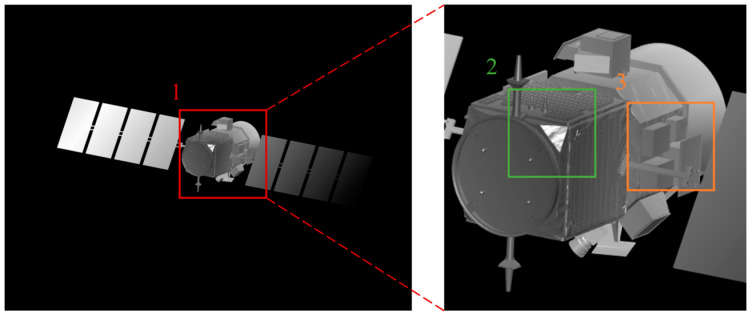
The results of laser 3D imaging (our methods).

In order to demonstrate the difference between the research method in this paper and the previous research methods, we simulated the two laser imaging results for comparison. Figure 18 shows the results of conventional laser imaging, and Figure 19 shows the results of laser 3D imaging using the laser scattering model established in this paper. In Figure 18 and Figure 19, the image on the right is a magnification of the image area labeled 1 on the left. Comparing the imaging details in the two images, the traditional imaging model is too idealistic in the imaging area labeled 2, and the grayscale information completely changes with the imaging distance. The imaging method proposed in this paper can reflect the influence of light on the imaging results, which is closer to the actual imaging results. In the imaging area marked 3, the imaging details of the imaging model proposed in this paper are clearer and the resolution is higher than that of the traditional model due to the presence of illumination. Therefore, from the perspective of imaging fit and imaging details, the imaging model constructed in this paper has great advantages.

**Figure 20:**
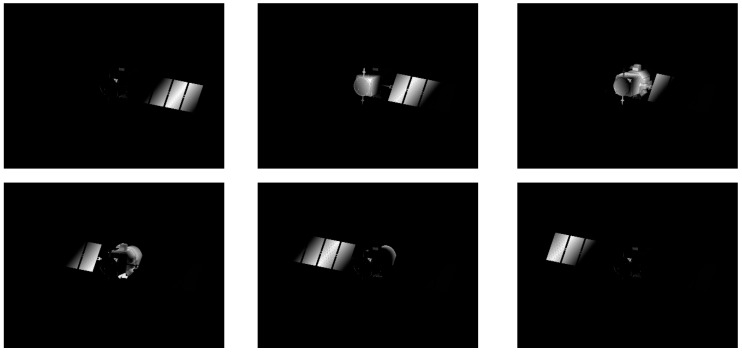
EO crystal modulation 2D imaging map.

The characteristics of laser 3D imaging are mainly related to the parameters of laser pulse width and gating shutter width. In the simulation experiment, the wavelength of the pulsed laser is 532 nm, the pulse energy is 200 mJ, the laser pulse width is 10 ns, the EO crystal modulation gate width is 10 ns, and the imaging distance is set to 10 km. The results of EO crystal modulation 2D imaging are shown in Figure 20.

**Figure 21:**
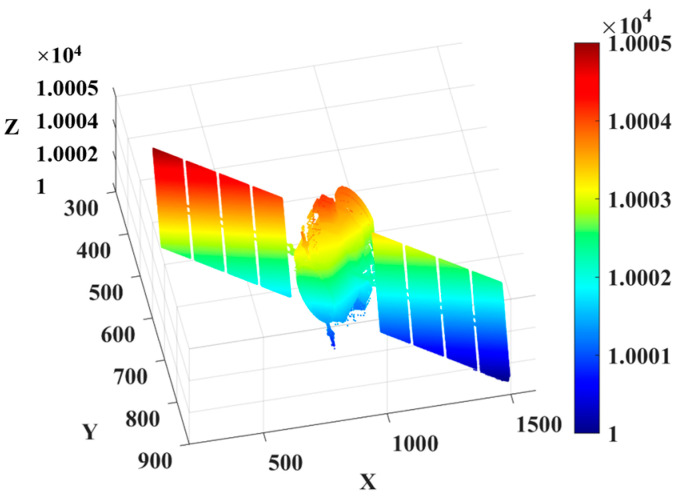
Three-dimensional point cloud recovery map of the imaging target.

In Figure 20, the electro–optical crystal gating result is a grayscale image, and the part of the object selected by the crystal shutter has grayscale information, so it can be intuitively observed that the simulation model can image the target at a specific distance and within a specific depth of field. The delay time of the EO crystal modulation is controlled, and the detector is gated from near to far until the imaging depth of field covers the entire target area. According to the principle of EO crystal modulation of three-dimensional imaging range information recovery, the target’s three-dimensional information is recovered using the two-dimensional grayscale image, and the three-dimensional point cloud of the satellite is shown in Figure 21.

In Figure 21, the *X* axis and *Y* axis represent the satellite’s pixel information captured by the camera, and the *Z* axis represents the satellite’s distance information. As the distance of the satellite increases, the color of the satellite 3D point cloud also changes gradually. On the right side of the 3D point cloud map is a color bar representing the distance, which can be used to judge the distance information of the satellite.

## 6. Conclusions

In this paper, we construct a simulation imaging model based on the principle of laser 3D imaging modulated by electro–optical crystals. The model focuses on the distribution of laser scattering characteristics of the imaging target scene, which makes the 3D imaging closer to the actual situation, provides a more accurate theoretical framework for subsequent research, and is helpful in understanding the physical mechanism of laser interaction with the target. Secondly, a five-parameter BRDF model of the target material is constructed, and a hybrid BBO–Firefly algorithm is proposed to fit the model parameters, which is significantly innovative in the field of laser scattering characteristics. Through experimental verification of gold foil and aluminum materials, the algorithm shows excellent performance in terms of fitting accuracy and iteration speed. Compared with the traditional algorithm, it can obtain the scattering characteristics of the target material more accurately, which provides a powerful technical means for the accurate description of the target characteristics in laser 3D imaging technology. Finally, we successfully verify the scientificity and reliability of the proposed imaging theory by conducting simulated imaging experiments on satellite targets at a distance of 10 km. The imaging results clearly present the three-dimensional information of the target, which indicates that the imaging method and related algorithms proposed in this study have good performance in practical applications. It provides a feasible technical solution for laser 3D imaging of long-distance targets, which has important theoretical and practical value, provides new ideas and methods for the development of related technologies, and is expected to promote further innovation and application in this field.

## 7. Patents

The relevant content of this article has applied for two national invention patents. The first patent name is a BRDF parameter fitting method and system for material surface based on lidar imaging, patent number ZL 2023 1 0531474.8. Another patent name is a three-dimensional imaging simulation modeling method and system for area array lidar, patent number ZL 2023 1 0531149.1.

## Figures and Tables

**Table 1:** Optimization results of the BBO algorithm, Firefly algorithm, and hybrid BBO–Firefly algorithm.

	$k_{b}$	$k_{d}$	$k_{r}$	a	b	MSE			
						15°	30°	45°	60°
**BBO–Firefly**	6.7790	0.0389	0.9479	0.8818	−38.8936	0.0636	0.0266	0.0485	0.0566
**BBO**	6.8690	0.0342	0.9471	0.9347	−34.1335	0.4253	0.3572	0.5103	0.5035
**Firefly**	6.9765	0.0358	0.8963	0.9439	−35.8712	0.3633	0.3244	0.4199	0.3765
**BBO–Firefly**	14.3215	0.0293	0.8364	0.7327	−45.6591	0.0478	0.0652	0.0598	0.0758
**BBO**	14.2614	0.0281	0.8166	0.6657	−41.4935	0.2885	0.3699	0.4826	0.6994
**Firefly**	15.3142	0.0278	0.7532	0.7118	−36.7565	0.3208	0.2733	0.2172	0.2262

## Data Availability

The data that support this study are proprietary in nature and may only be provided with restrictions.
